# Interface Bonding Properties of CrAlSiN-Coated Cemented Carbides Doped with CeO_2_ and Y_2_O_3_ Rare Earth Oxides

**DOI:** 10.3390/molecules28083584

**Published:** 2023-04-20

**Authors:** Junru Yang, Yanping Yue, Yan Wang, Yuekan Zhang

**Affiliations:** 1 College of Mechanical and Electronic Engineering, Shandong University of Science and Technology, Qingdao 266590, China; yangjunru@sdust.edu.cn (J.Y.); yue171442@163.com (Y.Y.); 2 Sinopec Qilu Company Ltd., Zibo 255400, China; 19862267401@163.com

**Keywords:** rare earth oxides, CrAlSiN/WC-Co cemented carbide, adhesion work, charge density difference, average Mulliken bond population

## Abstract

This study performed first-principle-based calculations of the interface adhesion work in interface models of three terminal systems: CrAlSiN_Si_/WC-Co, CrAlSiN_N_/WC-Co, and CrAlSiN_Al_/WC-Co. The results proved that the CrAlSiN_Si_/WC-Co and CrAlSiN_Al_/WC-Co interface models had the highest and lowest interface adhesion work values (4.312 and 2.536 J·m^−2^), respectively. Thus, the latter model had the weakest interface bonding property. On this basis, rare earth oxides CeO_2_ and Y_2_O_3_ were doped into the Al terminal model (CrAlSiN_Al_/WC-Co). Then, doping models of CeO_2_ and Y_2_O_3_ doped on the WC/WC, WC/Co, and CrAlSiN_Al_/WC-Co interfaces were established. The adhesion work value was calculated for the interfaces in each doping model. When CeO_2_ and Y_2_O_3_ were doped in the WC/WC and CrAlSiN_Al_/WC-Co interfaces, four doping models were constructed, each model contains interfaces withreduced adhesion work values, indicating deteriorated interface bonding properties. When the WC/Co interface was doped with CeO_2_ and Y_2_O_3_, the interface adhesion work values of the two doping models are both increased, and Y_2_O_3_ doping improved the bonding properties of the Al terminal model (CrAlSiN_Al_/WC-Co) more significantly than CeO_2_ doping. Next, the charge density difference and the average Mulliken bond population were estimated. The WC/WC and CrAlSiN_Al_/WC-Co interfaces doped with CeO_2_ or Y_2_O_3_, with decreased adhesion work, exhibited low electron cloud superposition and reduced values of charge transfer, average bond population, and interatomic interaction. When the WC/Co interface was doped with CeO_2_ or Y_2_O_3_, superposition of the atomic charge densities of electron clouds was consistently observed at the CrAlSiN_Al_/WC-Co interface in the CrAlSiN_Al_/WC/CeO_2_/Co and CrAlSiN_Al_/WC/Y_2_O_3_/Co models; the atomic interactions were strong, and the interface bonding strength increased. When the WC/Co interface was doped with Y_2_O_3_, the superposition of atomic charge densities and the atomic interactions were stronger than for CeO_2_ doping. In addition, the average Mulliken bond population and the atomic stability were also higher, and the doping effect was better.

## 1. Introduction

CrAlSiN-coated cemented carbide tools are widely known for their high hardness, corrosion resistance, wear resistance, and high-temperature oxidation resistance. These features have made them especially suitable for machining difficult-to-process materials, such as titanium alloys [1,2,3,4]. However, CrAlSiN-coated cemented carbide tools are more commonly associated with interface problems, such as coating disbondment and sticking [5]. Some scholars have doped additives into the coating or matrix to improve the interface properties of the coated cutting tools. For example, Lu et al. [6] prepared a diamond coating with a level of 8000 ppm doped B on the WC-Co cemented carbide matrix and found that, compared with undoped coated cutting tools, the residual stress was lower and the coating–matrix bonding strength was higher. Wang et al. [7] prepared a CrBN coating containing Ni or Cu doping on a 45 steel matrix. It was found that the coating hardness decreased after adding Ni or Cu, but the coating resistance to circular cracks was significantly enhanced due to good bonding properties between coating substrates. Yu et al. [8] utilized a cathodic arc evaporation system to prepare AlTiN coatings with various B/C doping ratios. The indentation method was used to evaluate the bonding strength between the coating and substrate. The results showed that the bonding strength between the coating and substrate was the best when the B/C ratio was 1:1.

Rare earth elements have high chemical activity and low electronegativity [9,10]. Extensive studies have been conducted worldwide on the reinforcement of material performance using rare earth element doping, which has been found to improve the interface bonding strength. Li et al. [11] employed the sol–gel method to prepare WC-10 (Co, x-Re) composite powders with various rhenium content levels. Microstructural analysis showed that adding rhenium resulted in a more regular WC grain shape and more equiaxed grains. In addition, the interface between the Co binder phase and WC was smooth and without pores, and the interface bonding was satisfactory. Liu et al. [12] reported that doping rare earth elements into WC-Co cemented carbides inhibited grain growth, refining the grains and increasing the interface bonding strength. Zou et al. [13] proved that doping with appropriate amounts of rare earth borides LaB_6_ and CeB_6_ purified the grain and phase boundaries and improved the WC/Co interface wettability, thereby increasing the interface bonding strength. Wang et al. [14] manufactured WC-11Co cemented carbides with and without CeO_2_ doping by vacuum hot-pressing sintering. Their study showed that CeO_2_ addition significantly improved the cutter’s fracture toughness. Wen [15] doped nano-CeO_2_ into WC-10Co cemented carbide and reported that CeO_2_ addition improved the fracture toughness of the cemented carbide. The reason was that CeO_2_ was enriched at the WC grain boundaries and reacted with impurity elements to purify the grain and phase boundaries. As a result, the wettability and bonding strength of the WC/Co interface were improved, finally increasing the grain and phase boundaries’ strength and the cemented carbide’s fracture toughness. Guo et al. [16] prepared a WC-6Co cemented carbide doped with Y_2_O_3_. During solid-phase sintering, the doped Y_2_O_3_ effectively inhibited the growth of WC grains, while Y_2_O_3_ located in the WC/Co grain boundary separated WC and Co, thereby inhibiting the dissolution and reprecipitation of WC and increasing the interface bonding strength. Wang et al. [17] doped Y_2_O_3_ into the WC-10Co cemented carbide. They found that the Y_2_O_3_ particles were pinned to the WC grain and phase boundaries, hindering the diffusion, dissolution, and growth of WC particles and hence refining the grains. Huang [18] used a wet grinding style to study the effects of CeO_2_ and Y_2_O_3_ doping on WC-10Co cemented carbides. The results showed that CeO_2_ and Y_2_O_3_ doping increased the fracture toughness of the cemented carbides from 12.8 MPa·m^1/2^ before doping to 16.7 MPa·m^1/2^ after doping. Yang et al. [19] employed spark plasma sintering (SPS) technology to prepare a WC-8Co-Y_2_O_3_ cemented carbide. It was found that Y_2_O_3_ addition increased the WC/Co interface strength, thereby improving the hardness and fracture toughness of the cemented carbide. Qin et al. [20] employed solid–liquid doping and SPS technology to prepare a Y_2_O_3_-doped WC-12Co cemented carbide. The results showed that the semicoherent interphase boundary between Y_2_O_3_ and WC increased the hardness and fracture toughness of the Y_2_O_3_-doped cemented carbide by 2.1 and 9.2%, respectively, compared with that before doping. Deng et al. [21] employed the in situ synthesis and spray drying process to prepare a CeO_2_-doped WC-10Co cemented carbide. Their study showed that CeO_2_ doping decreased the surface energy differences between the crystal planes while increasing the wettability of the interface between the Co binder phase and WC grains. For this reason, the degree of densification, hardness, and toughness of the cemented carbide were improved.

The above brief survey of existing studies in the relevant field reveals that most of them have focused on the performance of WC-Co cemented carbides doped with rare earth oxides and achieved this purpose experimentally. However, few studies have been conducted on the interface performance of CrAlSiN-coated cemented carbides. From an atomic perspective, there still needs to be more investigations into the interface bonding mechanism of CrAlSiN-coated cemented carbides doped with rare element oxides. Therefore, in this paper, based on the first-principles method, from the microscopic atomic point of view, the present study focused on the bonding performance of the coating–matrix interface in the interface models of CrAlSiN-coated WC-Co cemented carbides with different terminal atoms. First, we determined the interface model with the worst interface bonding performance. CeO_2_ and Y_2_O_3_ were doped into the WC/WC, WC/Co, and CrAlSiN_Al_/WC-Co interfaces of CrAlSiN-coated cemented carbides. The adhesion work was calculated for each interface model, and the charge density difference and average Mulliken bond population were estimated. On this basis, the influence pattern and the nature of the interface bonding performance of CrAlSiN-coated cemented carbides doped with rare earth oxides were revealed. These research findings are instrumental in the design optimization, popularization, and application of coated cemented carbide cutting tools with improved interface bonding properties.

## 2. Parameter Selection for Simulation Analysis and Parameter Calculation of Interface Bonding Properties

### 2.1. Parameter Selection for Simulation Analysis

The main research in this paper was carried out in the CASTEP module of the Materials Studio software. Geometric optimization was implemented for all models (primal cells such as WC, Co, CrN, Al, Si, CeO_2_, and Y_2_O_3_ and all models before and after doping) based on the first principle and density functional theory. The simulation parameters were chosen as follows.

Based on the Monkhorst–Pack algorithm, the k-point grid was set to 5×5×1. The energy of each unit cell was determined under different cutoff energies, and it was found that the energy of the unit cell tended to converge at the cutoff energy of 400 eV; therefore, Ecut was set to 400 eV. The structure of the original cell was optimized under different exchange correlation functions; we found that when the exchange association function is GGA-PBE, the calculated lattice constants of the optimized cell have the least deviation from the experimental values; therefore, GGA-PBE was selected as the exchange association function. Ultrasoft pseudopotential was chosen to describe the interactions between valence electrons and the nuclei of ions.

The Broyden–Fletcher–Goldfarb–Shanno (BFGS) algorithm was used to optimize the model. The optimization parameters were as follows: the SCF convergence threshold was specified as 1.0 × 10^−5^ eV/atom; the maximum interatomic interaction was 0.03 eV/Å; the maximum intracrystalline stress was 0.05 GPa; the maximum atomic displacement was 0.001 Å; and the number of iteration steps was 100 [22].

### 2.2. Parameter Calculation of Interface Bonding Properties

After the geometric optimization of the interface models, simulation calculation was conducted again using the above parameters to obtain the energy on surfaces α and β and the total energy and area of the α/β interface involved in the optimized interface models. Finally, the adhesion work at the interface was estimated using the relevant formula. Adhesion work is an important parameter characterizing interface bonding properties. It is defined as the reversible work required to separate two phases from each other. The higher the adhesion work, the stronger the interatomic bonding at the interface; hence, the stronger the interface bonding properties and the more stable the interface structure. The adhesion work at the interface between α and β can be calculated as follows [23]:

(1) $$W_{\mathrm{ad}}=\frac{E_{\alpha }+E_{\beta }-E_{\alpha /\beta }}{A}$$

where W_ad_ is the adhesion work, J/m^2^; E_α_ and E_β_ are the energies of surfaces α and β, eV; E_α/β_ is the total energy of the α/β interface system, eV; and A is the area of the interface, Å^2^.

## 3. Analysis of Interface Bonding Properties of the CrAlSiN/WC-Co Model Non-Doped with CeO_2_ or Y_2_O_3_

### 3.1. Construction of the CrAlSiN/WC-Co Model

#### 3.1.1. Construction of the WC-Co Cemented Carbide Matrix Model

**Figure 1:**
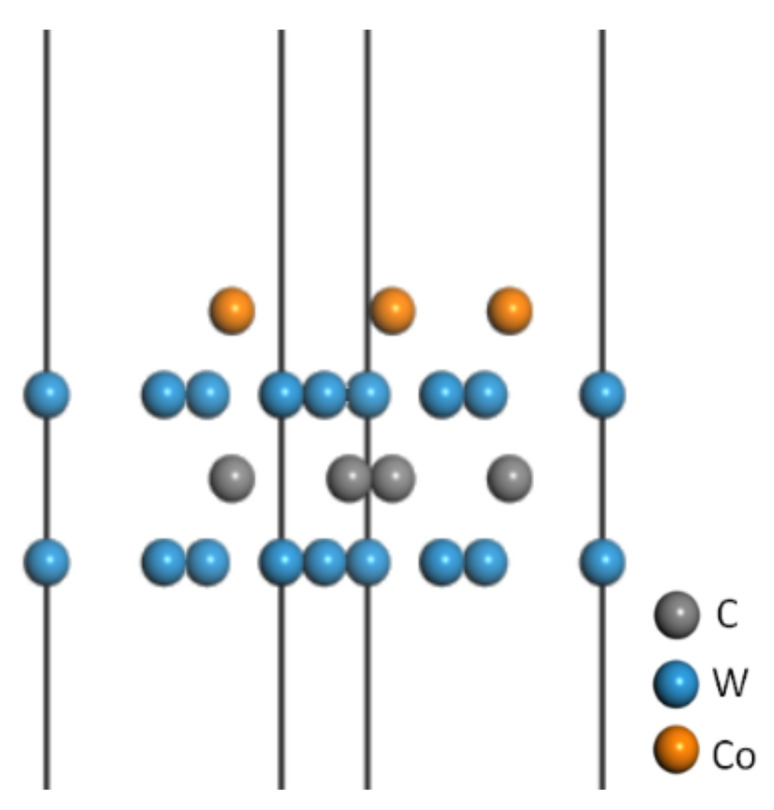
WC-Co model with a vacuum layer.

The WC(0001) crystal face with a W terminal atom is the most stable, and Co can replace the C atom on the WC(0001) surface [24,25]. Considering this, we used the Co atoms to replace the C atoms on the WC(0001) surface with W terminal atoms and added a 20 Å vacuum layer to build the WC-Co model with Co content of 10.4 wt%. As shown in Figure 1, this model was used to approximately represent the cemented carbide matrix YG10.

#### 3.1.2. Construction of the CrAlSiN Coating Model

**Figure 2:**
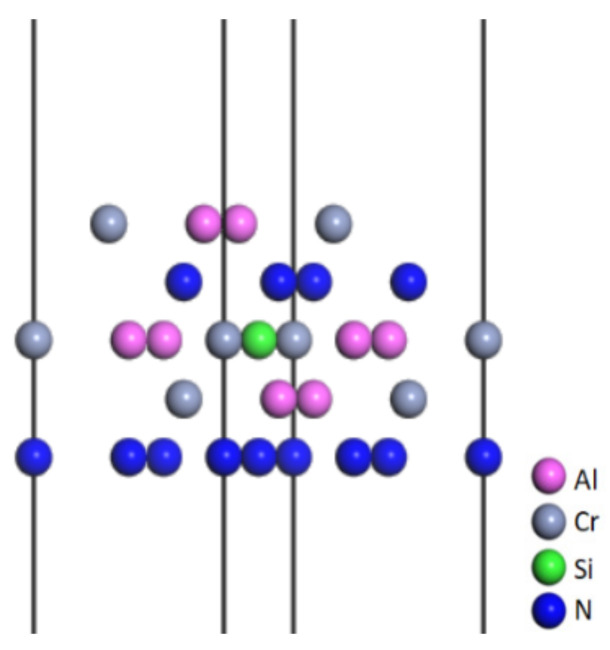
CrAlSiN model with a vacuum layer.

CrAlSiN and CrAlN grew preferably in the (111) orientation [26]. Al replaced some Cr atoms to become the solid solution in CrN. Si entered the CrAlSiN coating by replacing the Al atoms [27,28]. In addition, the hardness values of the CrAlN and CrAlSiN coatings were higher when Al and Si atoms accounted for 31 and 4.88 wt%, respectively [29,30]. Then, the CrAlN(111) model with Al content of 33 wt%. was built by replacing the Cr atoms in the supercell CrN(111) with Al atoms. Next, Si atoms were used to replace the Al atoms in CrAlN(111), and a 20 Å vacuum layer was added to obtain a CrAlSiN model with Si content of 4.9 wt%. Figure 2 shows a CrAlSiN_Al_ coating model with a vacuum layer.

#### 3.1.3. Construction of the CrAlSiN/WC-Co Models with Different Terminal Atoms

**Figure 3:**
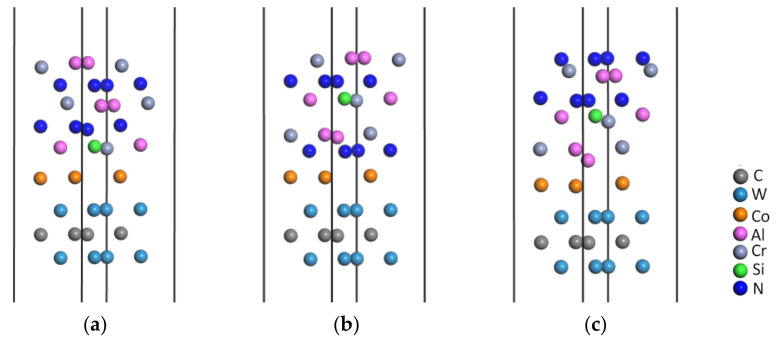
CrAlSiN/WC-Co model after geometric optimization: (**a**) CrAlSiN_Si_/WC-Co; (**b**) CrAlSiN_N_/WC-Co; (**c**) CrAlSiN_Al_/WC-Co.

The interface bonding strength between the WC-Co matrix and the CrAlSiN coating directly impacted the usability of CrAlSiN-coated cemented carbides. There are three terminal atoms on the CrAlSiN crystal surface: Si, N, and Al. Next, interface models with different terminal atoms were built using the WC-Co cemented carbide matrix and the CrAlSiN coating. The parameters selected for simulation analysis as above were used for the geometric optimization of the interface models Figure 3 shows the CrAlSiN/WC-Co models with Si, N, and Al terminal atoms after geometric optimization.

### 3.2. Interface Bonding Property Analysis

Based on the abovementioned parameters, the CrAlSiN/WC-Co models with three different terminal atoms after geometric optimization were used to calculate the total energy E_α/β_ on the two surfaces E_α_ and E_β_ and the area of the interface A_α/β_. The calculated results were substituted into Formula (1) to obtain the adhesion work at the interfaces, as shown in Table 1.

Table 1 shows that the adhesion work was the largest for the CrAlSiN_Si_/WC-Co model, with a value of 4.312 J·m^−2^. This model had the highest interface bonding strength and the most stable interface. The adhesion work was the smallest for the Al terminal model (CrAlSiN_Al_/WC-Co), with a value of 2.536 J·m^−2^. This model had the lowest interface bonding strength and the most unstable interface.

The Al terminal model (CrAlSiN_Al_/WC-Co), the most unstable model, was doped with rare earth oxides CeO_2_ and Y_2_O_3_ to build doped models. The adhesion work at this interface was calculated. The charge density difference and the average Mulliken bond population were analyzed. On this basis, we discussed the effects of doping rare earth oxides on the interface bonding properties of CrAlSiN-coated cemented carbides from the perspectives of charge transfer and bonding mode.

## 4. Analysis of the Interfaces Bonding Properties of the Al Terminal Model Doped with CeO_2_ or Y_2_O_3_

### 4.1. Construction of Doped Models

#### 4.1.1. Construction of the CeO_2_ and Y_2_O_3_ Models

**Figure 4:**
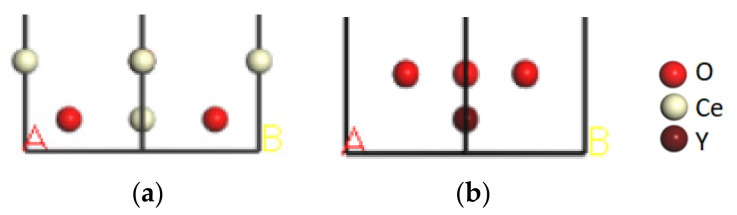
Rare earth oxide models with a vacuum layer: (**a**) CeO_2_(001); (**b**) Y_2_O_3_(001).

The CeO_2_(001) and Y_2_O_3_(001) crystal faces were the most stable. Considering the effects of atoms in the first two layers near the interface on the interface [31], we built the CeO_2_(001) and Y_2_O_3_(001) models with two layers and a 20 Å vacuum layer, A and B are used to show the direction of vacuum layer, as shown in Figure 4.

#### 4.1.2. Construction of Al Terminal Models Doped with CeO_2_ or Y_2_O_3_

**Figure 5:**
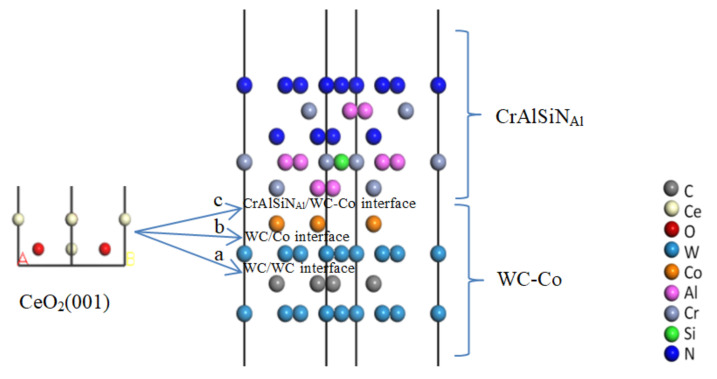
Construction of models doped with CeO_2_(001): (**a**) CrAlSiN_Al_/WC/CeO_2_/WC-Co model; (**b**) CrAlSiN_Al_/WC/CeO_2_/Co model; (**c**) CrAlSiN_Al_/CeO_2_/WC-Co model.

The modeling processes of CeO_2_(001) doping into the WC/WC, WC/Co, and CrAlSiN_Al_/WC-Co interfaces are shown in Figure 5a–c. The procedures for Y_2_O_3_ doping into the WC/WC, WC/Co, and CrAlSiN_Al_/WC-Co interfaces were the same as those for CeO_2_ doping.

### 4.2. Geometric Optimization of the Doped Models

**Figure 6:**
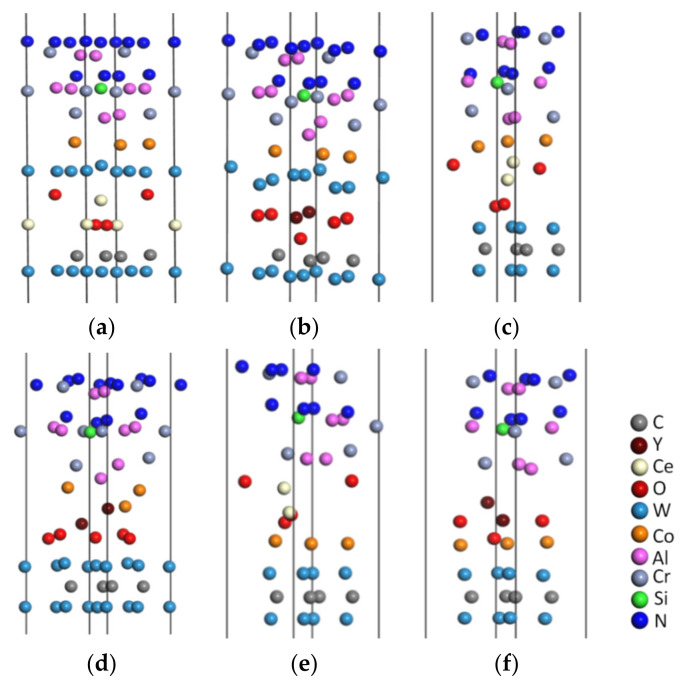
Doped models after geometric optimization: (**a**) CrAlSiN_Al_/WC/CeO_2_/WC-Co; (**b**) CrAlSiN_Al_/WC/Y_2_O_3_/WC-Co; (**c**) CrAlSiN_Al_/WC/CeO_2_/Co; (**d**) CrAlSiN_Al_/WC/Y_2_O_3_/Co; (**e**) CrAlSiN_Al_/CeO_2_/WC-Co; (**f**) CrAlSiN_Al_/Y_2_O_3_/WC-Co.

The Al terminal models doped with CeO_2_ and Y_2_O_3_ were subjected to geometric optimization using the above parameters for simulation analysis to obtain an interface model with a stable structure. Figure 6a,b depict the optimized models with CeO_2_ and Y_2_O_3_ doped into the WC/WC interface, respectively; Figure 6c,d present the optimized models with CeO_2_ and Y_2_O_3_ doped into the WC/Co interface, respectively; Figure 6e,f depict the optimized models with CeO_2_ and Y_2_O_3_ doped into the CrAlSiN_Al_/WC-Co interface, respectively.

### 4.3. Analysis of the Interface Bonding Properties of the Al Terminal Model Doped with CeO_2_ or Y_2_O_3_

#### 4.3.1. Adhesion Work Analysis

The models doped with CeO_2_ or Y_2_O_3_ all contained several interfaces. For example, in the CrAlSiN_Al_/WC/CeO_2_/Co model, there were three interfaces: CrAlSiN_Al_/WC-Co, CeO_2_/Co, and WC/CeO_2_. All interface adhesion work values were calculated, as shown in Table 2, compared with the adhesion work values at the CrAlSiN_Al_/WC-Co interface before doping. As long as the adhesion work value of any of the multiple interfaces included in the doping model was smaller than that of the CrAlSiN_Al_/WC-Co interface before doping, CeO_2_ or Y_2_O_3_ doping decreased the interface bonding properties of the Al terminal model. Only when the adhesion work of all interfaces in the doped model was greater than that of the CrAlSiN_Al_/WC-Co interface before doping could the doping of CeO_2_ or Y_2_O_3_ improve the interface bonding performance of the Al terminal model.

CeO_2_ was doped into the WC/WC, WC/Co, and CrAlSiN_Al_/WC-Co interfaces in the Al terminal model to obtain the CrAlSiN_Al_/WC/CeO_2_/WC-Co, CrAlSiN_Al_/WC/CeO_2_/Co, and CrAlSiN_Al_/CeO_2_/WC-Co models, respectively. Y_2_O_3_ was doped into the WC/WC, WC/Co, and CrAlSiN_Al_/WC-Co interfaces in the Al terminal model to obtain the CrAlSiN_Al_/WC/Y_2_O_3_/WC-Co, CrAlSiN_Al_/WC/Y_2_O_3_/Co, and CrAlSiN_Al_/Y_2_O_3_/WC-Co models, respectively. Table 2 shows the adhesion work calculated at different interfaces with and without doping of CeO_2_ and Y_2_O_3_ into the WC/WC, WC/Co, and CrAlSiN_Al_/WC-Co interfaces.

It can be inferred from Table 2 that when CeO_2_ and Y_2_O_3_ were, respectively, doped into the WC/WC interface to build two doped models, both contained the CrAlSiN_Al_/WC-Co interface, and their adhesion work values were smaller than those of the CrAlSiN_Al_/WC-Co interface before doping. That is, doping CeO_2_ or Y_2_O_3_ into the WC/WC interface reduced the interface bonding properties of the Al terminal model.

In all doped models built by doping CeO_2_ or Y_2_O_3_ into the WC/Co interface, the adhesion work at all interfaces was consistently higher than that at the CrAlSiN_Al_/WC-Co interface before doping. In CrAlSiN_Al_/WC/CeO_2_/Co, the adhesion work values at the CrAlSiN_Al_/WC-Co, CeO_2_/Co, and WC/CeO_2_ interfaces were 4.216, 3.235, and 4.615 J·m^−2^, respectively. In CrAlSiN_Al_/WC/Y_2_O_3_/Co, the adhesion work values at the CrAlSiN_Al_/WC-Co, Y_2_O_3_/Co, and WC/Y_2_O_3_ interfaces were 4.297, 3.982, and 4.724 J·m^−2^, respectively. The calculation results showed that doping CeO_2_ or Y_2_O_3_ into the WC/Co interface enhanced the interface bonding properties of the Al terminal model.

Of the two doped models, CrAlSiN_Al_/CeO_2_/WC-Co and CrAlSiN_Al_/Y_2_O_3_/WC-Co, formed by doping CeO_2_ or Y_2_O_3_ into the CrAlSiN_Al_/WC-Co interface, there were interfaces with reduced adhesion work values. In summary, doping CeO_2_ or Y_2_O_3_ into the CrAlSiN_Al_/WC-Co interface deteriorated the interface bonding properties of the Al terminal model.

**Figure 7:**
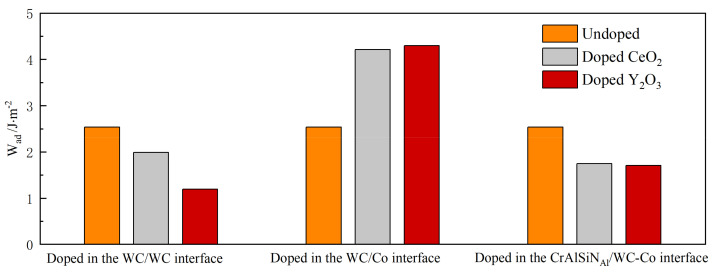
Comparison of the adhesion work for Al terminal model doped and not doped with CeO_2_ or Y_2_O_3_.

In order to compare the effects of doping CeO_2_ or Y_2_O_3_ at different interfaces of the Al terminal model on their interface bonding properties, we chose the adhesion work at the CrAlSiN_Al_/WC-Co interface among the WC/WC and WC-Co interfaces doped with CeO_2_ or Y_2_O_3_. The smallest adhesion work was chosen among the CrAlSiN_Al_/WC-Co interfaces doped with CeO_2_ or Y_2_O_3_. A comparison chart of the adhesion work was thus produced, as shown in Figure 7.

As shown in Figure 7, doping CeO_2_ or Y_2_O_3_ into the WC/WC and CrAlSiN_Al_/WC-Co interfaces reduced the adhesion work compared to non-doped interfaces—that is, doping impaired the interface bonding properties of the Al terminal model. Compared with the CrAlSiN_Al_/WC-Co interface adhesion work not doped with rare earth oxides, doping CeO_2_ or Y_2_O_3_ into the WC/Co interface increased the adhesion work—that is, doping improved the interface bonding properties of the Al terminal model. After doping rare element oxides into the WC/Co interface, further analysis revealed that of the two models, CrAlSiN_Al_/CeO_2_/WC-Co and CrAlSiN_Al_/Y_2_O_3_/WC-Co, the adhesion work at the CrAlSiN_Al_/WC-Co interfaces was 4.216 and 4.297 J·m^−2^, respectively. The increase was 1.68 and 1.761 J·m^−2^, respectively, compared with those before doping. Compared with CeO_2_ doping, Y_2_O_3_ doping more significantly improved the bonding properties for the Al terminal model.

#### 4.3.2. Charge Density Difference Analysis

Geometric optimization of the doped models caused charge redistribution among the atoms. The charge density difference maps allowed for the more intuitive observation of interatomic bonding in the system. In addition, the polarity of interatomic bonds could be assessed based on the spatial distribution of charge aggregation and charge transfer. Therefore, the interface bonding properties were characterized by the bonding strength.

**Figure 8:**
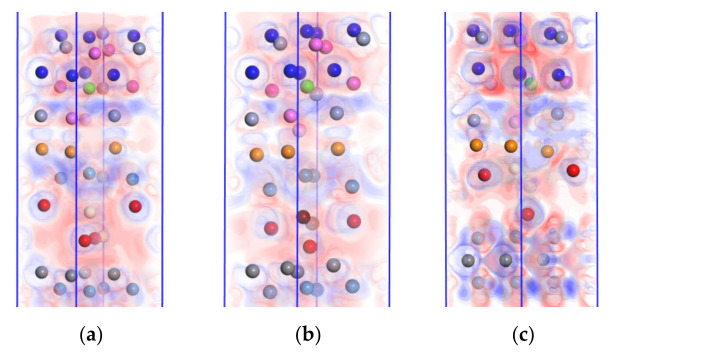

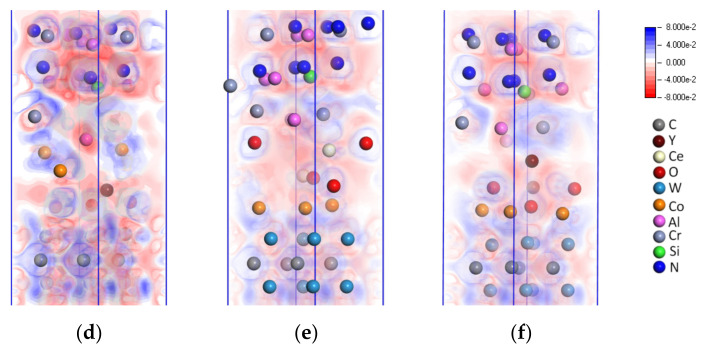
Charge density difference maps after doping CeO_2_ or Y_2_O_3_ into the WC/WC, WC/Co, and CrAlSiN_Al_/WC-Co interfaces: (**a**) CrAlSiN_Al_/WC/CeO_2_/WC-Co; (**b**) CrAlSiN_Al_/WC/Y_2_O_3_/WC-Co; (**c**) CrAlSiN_Al_/WC/CeO_2_/Co; (**d**) CrAlSiN_Al_/WC/Y_2_O_3_/Co; (**e**) CrAlSiN_Al_/CeO_2_/WC-Co; (**f**) CrAlSiN_Al_/Y_2_O_3_/WC-Co.

The charge density difference was calculated for the doped models after geometric optimization, with the results shown in Figure 8, where the regions with electron loss, gain, and zero transfer are indicated by red, blue, and white colors, respectively.

Figure 8a,b present the charge density difference maps after doping CeO_2_ or Y_2_O_3_ into the WC/WC interface, where regions without electrons or with low charge density existed at the CrAlSiN_Al_/WC-Co interface. This result indicated that doping CeO_2_ or Y_2_O_3_ into the WC/WC interface decreased the interatomic interactions between the CrAlSiN_Al_ coating and the WC-Co matrix, thus lowering the interface bonding properties.

Figure 8c,d depict the charge density difference maps after doping CeO_2_ or Y_2_O_3_ into the WC-Co interface. As shown in Figure 8c, the interatomic distance decreased at the CrAlSiN_Al_/WC-Co interface while the superposition of charge densities was enhanced. This phenomenon was more pronounced in the blue region near the Co atom, indicating the loss of many charges. Charge aggregation was more pronounced near the Cr and Al atoms, resulting in the gain of many charges. As a result, atoms at the CrAlSiN_Al_/WC-Co interface exhibited strong covalency, and the interface bonding strength increased. At the WC/CeO_2_/Co interface in (c), charge transfer in Ce-W and O-Co atom pairs was significant, accompanied by increased charge density, enhanced interatomic interactions, high covalency, and high interface bonding strength. At the CrAlSiN_Al_/WC-Co interface in (d), the atomic positions were changed, and charge transfer occurred between Al, Cr, and Co atoms. Interatomic interactions and covalency were strengthened, being manifested as increased bonding strength at the CrAlSiN_Al_/WC-Co interface. At the WC/Y_2_O_3_/Co interface in (d), the high charge density in the O-W atom pair suggested strong attraction in this atom pair and high interface bonding strength.

Figure 8e,f present the charge density difference maps after doping CeO_2_ and Y_2_O_3_ into the CrAlSiN_Al_/WC-Co interface, respectively. In the CrAlSiN_Al_/CeO_2_/WC-Co model, the electron clouds were superposed between Al, O, and Ce atoms, leading to strong interatomic interactions. Charge transfer occurred in the Co-O atom pair at the interface. There were shared charges between Co-O and Ce atoms, accompanied by decreased charge density and weak interatomic interactions. In the CrAlSiN_Al_/Y_2_O_3_/WC-Co model, O and Y atoms moved away from the CrAlSiN_Al_ coating. There was a greater distance between Al and O atoms, less superposition of electron clouds, and lower interatomic interactions. In contrast, the superposition of electron clouds was greater between the Co atom and O-Y, which meant stronger interatomic interactions. That is, doping CeO_2_ or Y_2_O_3_ into the CrAlSiN_Al_/WC-Co interface deteriorated the interface bonding properties of the Al terminal model. The results of the charge density difference analysis agreed with those of the adhesion work analysis.

#### 4.3.3. Mulliken Average Bond Population Analysis

**Figure 9:**
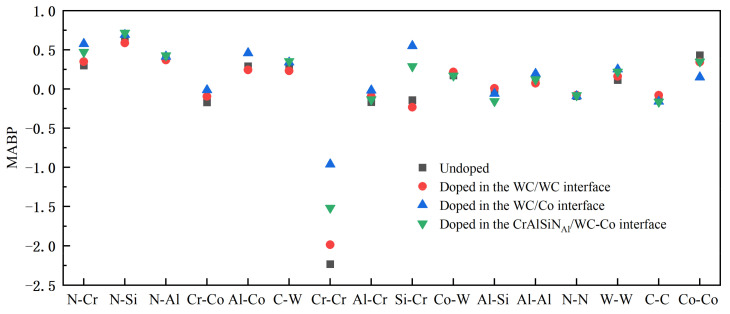
MABPs at different interfaces doped and not doped with CeO_2_.

**Figure 10:**
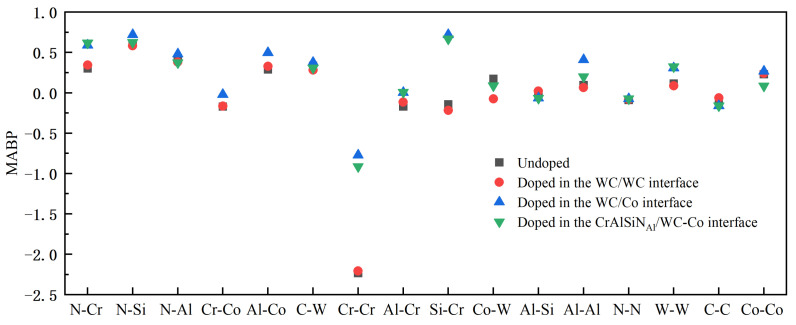
MABPs at different interfaces doped and not doped with Y_2_O_3_.

**Figure 11:**
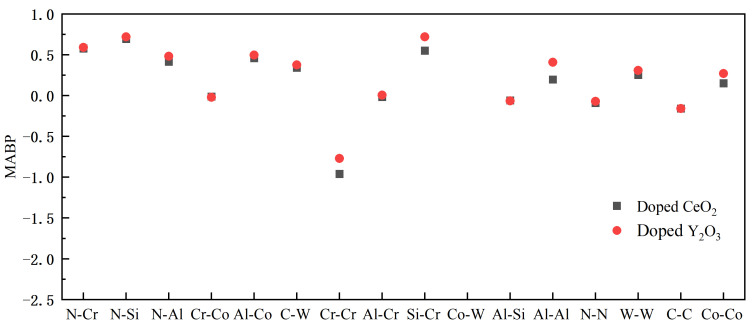
Comparison of MABPs at different interfaces doped with CeO_2_ or Y_2_O_3_.

Mulliken bond population (MBP) analysis is a widely used method to calculate atomic charges. The MBP characterizes the interatomic bonding strength. A positive MBP usually indicates covalency; the higher the value, the stronger the covalency and the interatomic interactions. A negative MBP indicates antibonding, and the higher the absolute value, the more unstable the bonding, the smaller the interatomic interactions, and the greater the repulsion. Calculating the Mulliken average bond population (MABP) allows one to analyze the interatomic bonding strength better. The MABPs were calculated at the WC/WC, WC/Co, and CrAlSiN_Al_/WC-Co interfaces doped with CeO_2_ and Y_2_O_3_, as shown in Figure 9, Figure 10 and Figure 11.

Figure 9 compares the MABPs between different atoms at the WC/WC, WC/Co, and CrAlSiN_Al_/WC-Co interfaces doped and not doped with CeO_2_. It is easy to see that when the MABP was above zero, doping CeO_2_ into the WC/WC and CrAlSiN_Al_/WC-Co interfaces did not dramatically increase the MABP. In addition, the MABP was generally higher if CeO_2_ was doped into the WC-Co interface than if CeO_2_ was not doped or doped into the WC/WC and CrAlSiN_Al_/WC-Co interfaces. Increased MABP values indicated stronger covalency and higher interatomic forces. At negative MABP values, their absolute magnitudes were smallest when CeO_2_ was doped into the WC/Co interface, as shown by the comparison between the four data groups. This indicated that doping CeO_2_ into the WC-Co interface more significantly reduced interatomic repulsion. Compared with no doping or doping into the WC/WC and CrAlSiN_Al_/WC-Co interfaces, doping CeO_2_ into the WC/Co interface led to higher interatomic stability. In other words, doping CeO_2_ into the WC/Co interface improved the interface bonding strength more significantly in the Al terminal model.

Figure 10 compares the MABPs between different atoms after doping Y_2_O_3_ into the WC/WC, WC/Co, and CrAlSiN_Al_/WC-Co interfaces. As shown by the figure, positive MABPs were generally higher after doping Y_2_O_3_ into the WC/Co interface than after not doping or doping Y_2_O_3_ into other interfaces. This result indicated that doping Y_2_O_3_ into the WC/Co interface significantly increased the interatomic forces, strengthening the interatomic bonding. When the MABPs were negative, the absolute values after doping Y_2_O_3_ into the WC/Co interface were generally smaller than those after not doping or doping Y_2_O_3_ into other interfaces. This result indicated that doping Y_2_O_3_ into the WC/Co interface led to higher interatomic stability than not doping or doping into other interfaces. In the meantime, the interatomic repulsion decreased, and the interface bonding properties were improved more significantly in the Al terminal model.

Figure 11 compares the MABPs after doping Y_2_O_3_ or CeO_2_ into the WC/Co interface. When the MABP was positive, doping Y_2_O_3_ resulted in a higher MABP than doping CeO_2_. When the MABP was negative, the absolute value after doping Y_2_O_3_ was smaller than that after doping CeO_2_. The above results indicated that Y_2_O_3_ doping more significantly improved the interatomic bonding and interatomic forces than doping CeO_2_. For this reason, doping Y_2_O_3_ into the WC/Co interface more dramatically improved the interface bonding properties of the Al terminal model.

In summary, the charge density difference analysis and MBP analysis results agreed with those of the adhesion work analysis. There is good consistency between these results and those of available experimental studies.

In particular, Wen [15] prepared a CeO_2_-doped WC-10Co cemented carbide using a gas-pressure sintering furnace. The WC grain size and fracture toughness were measured in the cemented carbides doped and not with CeO_2_ using a Scanning Electron Microscope (SEM) and the press-in method, respectively. Their results showed that in the CeO_2_-doped cemented carbide, the average WC grain size decreased from 530 to 410 nm after CeO_2_ doping. This means that CeO_2_ doping refined the WC grains. Moreover, the fracture toughness of the CeO_2_-doped cemented carbide increased from 9.32 to 10.6 MPa·m^1/2^ after doping. The reason was that CeO_2_ was enriched at the WC grain boundaries, reacting with impurity elements to purify the grain and phase boundaries. As a result, the wettability of the WC/Co interface was enhanced, as was the bonding strength of the WC/Co interface. This result agrees with our finding that doping CeO_2_ into the WC/Co interface improved the bonding properties in the Al terminal model.

Wang et al. [17] prepared a Y_2_O_3_-doped WC-10Co cemented carbide using vacuum sintering. The cemented carbide’s surface morphology and Rockwell hardness were measured by SEM and the press-in method before and after doping, respectively. The bending strength of the cemented carbides was determined using an electronic universal testing machine. The analysis revealed that Y_2_O_3_ doping decreased the WC grain size in the cemented carbide, thus refining the grains. Due to its high affinity, Y_2_O_3_ reacted with the impurities at the grain boundaries, transforming the existing form of the impurities and improving the bonding strength of the WC/Co phase. As a result, the hardness of the cemented carbide increased from HRA 92.3 before doping to HRA 94.5 after doping. The bending strength increased from 1988 MPa before doping to 2250 MPa after doping. These results agree with our finding that doping Y_2_O_3_ into the WC/Co interface improved the bonding properties in the Al terminal model.

Huang [18] prepared WC-10Co cemented carbides doped with Y_2_O_3_ and CeO_2_ using a wet grid style. The surface morphology of cemented carbides before and after rare earth oxide doping was observed by SEM. The results showed that the WC grains in the cemented carbides doped with rare earth oxides were more rounded than those in non-doped cemented carbides. This was because the rounded WC grains had a larger contact area with Co, which affected the mechanical performance of the cemented carbides. The fracture toughness of the cemented carbides was determined using the Palmqvist toughness test. The fracture toughness of the Y_2_O_3_-doped cemented carbide (16.7 MPa·m^1/2^) was found to be larger than that of the CeO_2_-doped cemented carbide (15.2 MPa·m^1/2^). In addition, the fracture toughness of Y_2_O_3_- or CeO_2_-doped cemented carbides exceeded that of non-doped ones (12.8 MPa·m^1/2^). These results confirm our finding that doping Y_2_O_3_ into the CrAlSiN/WC-Co interface model improved the bonding properties in the Al terminal model.

## 5. Conclusions

(1) The adhesion work values were calculated for three interface models with various terminal atoms, namely CrAlSiN_Si_/WC-Co, CrAlSiN_N_/WC-Co, and CrAlSiN_Al_/WC-Co. The analysis showed that the adhesion work was the highest at the CrAlSiN_Si_/WC-Co interface (4.312 J·m^−2^) and the lowest at the CrAlSiN_Al/_WC-Co interface (2.536 J·m^−2^).
(2) Based on the CrAlSiN_Al/_WC-Co interface model with the lowest interface bonding strength, we doped CeO_2_ or Y_2_O_3_ into the WC/WC, WC/Co, and CrAlSiN_Al_/WC-Co interfaces to obtain the doped models.
(3) Doping CeO_2_ or Y_2_O_3_ into the WC/WC and CrAlSiN_Al_/WC-Co interfaces deteriorated the interface bonding properties of the Al terminal model; in contrast, doping into the WC/Co interface improved the bonding properties of the Al terminal model. Doping either CeO_2_ or Y_2_O_3_ into the WC/Co interface increased the adhesion work. Further charge density difference and MABP analyses revealed that the interfaces with higher adhesion work and improved interface bonding properties exhibited a decreased interatomic distance, a higher charge density, a larger number of charge transfers between atoms, stronger interatomic interactions, a higher MABP, and higher interatomic bonding strength.
(4) Of the two rare earth oxides, Y_2_O_3_ doping into the WC/Co interface improved the interface bonding properties more significantly than CeO_2_ doping. In CrAlSiN_Al_/WC/CeO_2_/Co, the adhesion work at the CrAlSiN_Al_/WC-Co, CeO_2_/Co, and WC/CeO_2_ interfaces was 4.216, 3.235, and 4.615 J·m^−2^, respectively. In CrAlSiN_Al_/WC/Y_2_O_3_/Co, the adhesion work values at the CrAlSiN_Al_/WC-Co, Y_2_O_3_/Co, and WC/Y_2_O_3_ interfaces were 4.297, 3.982, and 4.724 J·m^−2^, respectively. The adhesion work with Y_2_O_3_ doping was consistently higher than that with CeO_2_ doping. The constructed charge density difference maps revealed that Y_2_O_3_ doping into each interface consistently resulted in a higher charge density, a higher number of charge transfers, and stronger interatomic interactions. The MABP of the Y_2_O_3_-doped models was consistently higher than that of the CeO_2_-doped models. These results strongly suggested that Y_2_O_3_ doping more significantly increased the interatomic interactions and reduced the interatomic repulsion in the Al terminal model (CrAlSiN_Al_/WC-Co) compared to CeO_2_ doping. Therefore, when rare earth oxides are doped at the WC/Co interface, the doping of Y_2_O_3_ has a better effect in terms of improving the interface bonding performance of the Al terminal model (CrAlSiN_Al_/WC-Co).

## Figures and Tables

**Table 1:** Adhesion work of the CrAlSiN/WC-Co model.

Model	E_α_/eV	E_β_/eV	E_α/β_/eV	A_α/β_/Å^2^	W_ad_/J·m^−2^
CrAlSiN_Si_/WC-Co	14,954.098	19,210.462	−34,172.070	27.868	4.312
CrAlSiN_N_/WC-Co	14,950.463	19,210.372	−34,167.178	27.411	3.702
CrAlSiN_Al_/WC-Co	14,953.219	19,209.751	−34,167.157	26.418	2.536

**Table 2:** Adhesion work at different interfaces with and without CeO_2_ or Y_2_O_3_ doping.

Doping Type	Interface Model	Interface	E_α_/(eV)	E_β_/(eV)	E_α/β_/(eV)	A_α/β_/(Å^2^)	W_ad_/(J·m^−2^)
Undoped	CrAlSiN_Al_/WC-Co	CrAlSiN_Al_/WC-Co	−14,953.219	−19,209.751	−34,167.157	26.418	2.536
Doped CeO_2_	CrAlSiN_Al_/WC/ CeO_2_/WC-Co	CrAlSiN_Al_/WC-Co	−14,952.436	−23,075.467	−38,031.572	29.494	1.990
		WC-Co/CeO_2_	−25,809.872	−12,214.276	−38,031.572	29.494	4.027
	CrAlSiN_Al_/WC/ CeO_2_/Co	CrAlSiN_Al_/WC-Co	−82,179.012	−15,042.382	−97,229.165	29.494	4.216
		CeO_2_/Co	−78,884.118	−18,339.084	−97,229.165	29.494	3.235
		WC/CeO_2_	−74,637.363	−22,583.294	−97,229.165	29.494	4.615
	CrAlSiN_Al_/CeO_2_/ WC-Co	CrAlSiN_Al_/CeO_2_	−15,041.256	−82,183.017	−97,229.200	29.494	2.673
		CeO_2_/WC-Co	−77,935.625	−19,290.353	−97,229.200	29.494	1.748
Doped Y_2_O_3_	CrAlSiN_Al_/WC/ Y_2_O_3_/WC-Co	CrAlSiN_Al_/WC-Co	−14,952.029	−20,895.258	−35,849.532	30.051	1.195
		WC-Co/Y_2_O_3_	−25,808.481	−10,034.951	−35,849.532	30.051	3.248
	CrAlSiN_Al_/WC/ Y_2_O_3_/Co	CrAlSiN_Al_/WC-Co	−20,905.148	−14,955.857	−35,869.075	30.051	4.297
		Y_2_O_3_/Co	−18,082.653	−17,778.943	−35,869.075	30.051	3.982
		WC/Y_2_O_3_	−16,078.979	−19,781.223	−35,869.075	30.051	4.724
	CrAlSiN_Al_/Y_2_O_3_/ WC-Co	CrAlSiN_Al_/Y_2_O_3_	−14,956.840	−20,909.197	−35,869.241	30.051	1.706
		Y_2_O_3_/WC-Co	−19,207.037	−16,652.194	−35,869.241	30.051	5.330

## Data Availability

Data sharing not applicable.

## Sample Availability

No samples were involved in this study.
